# Strengthening global preparedness and response to arboviral disease threats: a call to action

**DOI:** 10.1016/S1473-3099(25)00686-3

**Published:** 2025-11-24

**Authors:** WHO Global Arbovirus Initiative Technical Advisory Group, Tammy Allen, Samuel K. Dadzie, D. S. A. F. Dheerasinghe, Gamou Fall, N. R. Faria, Emilie Javelle, Maria Eugenia Grillet, Maria G Guzman, Kleber Giovanni Luz, Hmooda Toto Kafy, Ehsan Mostafavi, Lee-Ching Ng, Chantal Reusken, Thomas W. Scott, Naveen Rai Tuli, Marietjie Venter

**Affiliations:** 1 College of Public Health, Medical and Veterinary Sciences, James Cook University, Cairns, Queensland, Australia; 2 Parasitology Department, Noguchi Memorial Institute for Medical Research, University of Ghana, Accra, Ghana; 3 National Dengue Control Unit, Ministry of Health, Colombio, Sri Lanka; 4 Département de Virologie, Institut Pasteur de Dakar, Dakar, Senegal; 5 MRC Centre for Global Infectious Disease Analysis, School of Public Health, Imperial College London, London, United Kingdom; 6 Faculdade de Medicina, Instituto Medicina Tropical, Universidade de São Paulo, São Paulo, Brazil; 7 Unité Parasitologie et Entomologie, Département Risques Vectoriels, Institut de Recherche Biomédicale des Armées, Marseille, France; 8 Laboratorio de Biología de Vectores y Parásitos, Instituto de Zoología y Ecología Tropical, Facultad de Ciencias, Universidad Central de Venezuela, Caracas, Venezuela; 9 Institute of Tropical Medicine "Pedro Kouri", WHO/PAHO Collaborating Center for the Study of Dengue and Its Control, La Lisa, Cuba; 10 Departamento de Infectologia, Universidade Federal do Rio Grande do Norte, Natal, Brazil; 11 Directorate General of Primary Health Care, Federal Ministry of Health, Khartoum, Sudan; 12 WHO Collaborating Centre for Vector-Borne Diseases, Department of Epidemiology and Biostatistics, Research Centre for Emerging and Reemerging Infectious Diseases, Pasteur Institute of Iran, Tehran, Iran; 13 Environmental Health Institute, National Environment Agency, Singapore; 14 Centre for Infectious Disease Control, National Institute for Public Health and the Environment, Bilthoven, The Netherlands; 15 Department of Entomology and Nematology, University of California, Davis, California, United States of America; 16 South Delhi Municipal Corporation, Delhi, India; 17 Division of Emerging Viral Threats, One Health Surveillance and Vaccines; and Infectious Disease and Oncology Research Institute, University of the Witwatersrand, Johannesburg, South Africa

Arthropod-borne viruses (arboviruses), particularly those transmitted by *Aedes aegypti* and *Aedes albopictus*, are a growing global health threat. Approximately 70% of the world’s population is at risk of infection from dengue, chikungunya, Zika, and yellow fever viruses,^1^ with the burden rising sharply in recent years. This increasing risk is driven by a confluence of factors, including rapid and often unplanned urbanisation, climate change, and increasing interconnectedness through global travel and trade.

The expansion of urban environments has created ideal breeding grounds for *Aedes* mosquitoes, while climate change is extending transmission seasons, expanding vector ranges, decreasing vector extrinsic incubation periods, and increasing the frequency and magnitude of arboviral outbreaks. By 2050, it is projected that 65% of the global population will reside in urban areas, further amplifying the risk of arboviral transmission.^2^ The Intergovernmental Panel on Climate Change has highlighted the profound effect of rising temperatures and altered precipitation patterns on vector ecology, with direct consequences for arboviral disease dynamics.^3^

Past epidemics are stark reminders of the explosive potential of arboviruses. The 2005–06 chikungunya virus epidemic in the Indian Ocean region resulted in millions of cases, and the introduction of chikungunya virus in the Americas in 2013–14 led to over 3·7 million cases across 45 countries and territories.^4^ The Zika virus epidemic, which began in the Pacific in 2007 and subsequently swept through the Americas, caused an estimated 132 million cases, with devastating consequences, including congenital Zika syndrome in fetuses or infants and neurological complications in adults, such as Guillain-Barré syndrome.^5^ The recognition of sexual transmission of Zika virus is also cause for concern. Yellow fever has also re-emerged as a substantial threat, with large outbreaks in Angola (2015–16) and Brazil (2016–18) outstripping vaccine supplies and leading to the use of fractional dosing strategies.

Despite concerted efforts to control *Ae aegypti*, 2024 saw a record 14 million dengue cases reported across 134 countries and territories, including more than 52 000 severe cases and more than 10 000 deaths.^6^ Particularly concerning are the nine-fold increase of dengue virus infections in Africa since 2019,^7^ and the unprecedented rise in autochthonous dengue cases within temperate regions in recent years, notably in France and Italy.^8^ The global economic burden of dengue was estimated to be US$19 billion in 2022.^9^

Beyond *Aedes*-borne viruses, other arboviruses are emerging or re-emerging in new areas. The emergence of a novel Oropouche virus reassortment strain, detected in 2024, is associated with major outbreaks in the Americas, particularly in Brazil and Cuba,^10^ coinciding with the 2024 dengue emergency in the continent. Oropouche virus has been linked to congenital defects and neuroinvasive disease, echoing the severe outcomes observed during the Zika epidemic. West Nile virus continues to cause outbreaks in the USA, Africa, and Europe, and Japanese encephalitis virus remains a persistent threat in Asia and Australia.

In response to these growing challenges, WHO launched the Global Arbovirus Initiative (GLAI) in March, 2022.^11^ The GLAI provides a comprehensive, integrated framework built upon six strategic pillars: monitoring risk and anticipating outbreaks, reducing epidemic risk, strengthening vector control, enhancing prevention and epidemic preparedness, fostering innovation in diagnostics and interventions, and building a coalition of partners and stakeholders to mobilise resources. The initiative emphasises cross- sectoral collaboration and alignment with key WHO programmes, including the Eliminating Yellow Fever Strategy, the Global Vector Control Response, the Health Emergencies Programme, the Global Neglected Tropical Diseases Programme, the Special Programme for Research and Training in Tropical Diseases, and the International Pathogen Surveillance Network.

Since its inception, the GLAI has made substantial progress. A global dengue dashboard now enables real- time risk monitoring and lineage tracking (see appendix), and new toolkits for preparedness and risk communication have been developed to support pandemic readiness and response. Updated clinical management guidelines for arboviral diseases have also been published, providing crucial support for frontline health workers. However, important gaps remain, particularly in settings where basic diagnostic and surveillance infrastructure is scarce, now exacerbated by recent external funding cuts.

Innovation and technological advancement are central in the fight against arboviral diseases. Immunisation remains a cornerstone of prevention, exemplified by the highly effective live attenuated yellow fever 17D vaccine, which averted an estimated 24 000 deaths in 2018 alone,^12^ and is expected to protect a total of 1 billion people in high-risk settings by 2026.^13^ Key advances in dengue vaccine development include TAK-003 (Qdenga, Takeda, Singen, Germany) and Butantan-DV (Butantan Institute and National Institutes of Health, São Paulo, Brazil). TAK-003 is currently the only vaccine recommended by WHO for dengue prevention in children aged 6–16 years residing in high-transmission settings. Advances in chikungunya vaccine development include the live attenuated VLA1553 (IXCHIQ, Valneva, Saint Herblain, France) and the virus-like particle vaccine VIMKUNYA (Bavarian Nordic, Hellerup, Denmark). Although IXCHIQ was the first vaccine to gain US Food and Drug Administration approval in late 2023, its US license was suspended in August, 2025, due to safety concerns. VIMKUNYA received US and EU approval in early 2025. WHO is currently reviewing evidence to inform recommendations. No approved vaccines or monoclonal antibodies are currently available for Zika virus.^14^ These developments offer further opportunities to reduce the burden of arboviral illnesses. Yet, challenges persist in ensuring equitable access, maintaining high vaccination coverage amid growing vaccine hesitancy, and mounting rapid deployment during outbreaks.

Vector-control strategies are also evolving. Innovative approaches such as the release of *Wolbachia*- infected mosquitoes and the use of sterile insect techniques, targeted indoor residual spraying, and spatial repellents have shown substantial reductions in dengue incidence – up to 77% in pilot regions.^15^ Modelling-based early warning systems, including for climate-sensitive diseases, have shown promise in outbreak detection and preparedness and are now implemented across multiple countries and regions. However, accessible and validated climate-health tools remain scarce, predominantly focused on malaria, and largely developed in high-income countries, with few intuitive user interfaces, and limited operationalisation with local co-development.^16^

The exponential growth of arboviral genomic data is another key advancement, enabling improved vaccine and diagnostic design, complementing traditional surveillance, and enhancing our understanding of virus evolution and transmission dynamics in near real time. Despite large investments in genomic surveillance during the COVID-19 pandemic, its re-deployment for arboviruses requires sustainable investments in laboratory capacity and workforce training and retention.

Strengthening health systems is paramount. This includes integrating epidemiological, entomological, laboratory, and ecological data (including animal surveillance); enhancing disease reporting systems; and improving clinical care through personnel training, expanded diagnostics, and standardised management protocols. Community engagement is equally vital; locally accepted, culturally appropriate, and empowering interventions are more likely to be sustained and succeed in reducing disease risk.

There are substantial opportunities to strengthen prevention and control strategies, but their success depends on sustained investment, coordinated surveillance, robust institutional frameworks, and clear prioritisation.^17^ The international community—policy makers, health organisations, funders, researchers, and communities—must urgently mobilise resources to close key gaps in early detection, surveillance, response, and control, thereby reducing the risk of both current and future arboviral outbreaks. With the global threat of arboviruses escalating, the time for action is now.

## Supplementary Material

Appendix
