# Supplementary material for: Strengthening global preparedness and response to arboviral disease threats: a call to action
Source: Lancet Infect Dis. Author manuscript; Available in PMC 2026 Jan 1. (PMC7618433; doi:10.1016/S1473-3099(25)00686-3)
Supplement: Appendix [file EMS211304-supplement-Appendix.pdf]

Appendix

Author list and affiliations:

WHO Global Arbovirus Initiative Technical Advisory Group (alphabetic order):

Allen, Tammy[1], Dadzie, Samuel K.[2], Dheerasinghe, D. S. A. F.[3], Fall, Gamou[4], Faria, N. R.[5,6], Javelle, Emilie[7], Grillet, Maria Eugenia[8], Guzman, Maria G[9], Giovanni Luz, Kleber[10], Kafy, Hmooda Toto[11], Mostafavi, Ehsan[12], Ng, Lee-Ching[13], Reusken, Chantal[14], Scott, Thomas W.[15], Tuli, Naveen Rai[16], Venter, Marietjie[17]

1. College of Public Health, Medical and Veterinary Sciences, James Cook University, Cairns, Queensland, Australia.
2. Parasitology Department, Noguchi Memorial Institute for Medical Research, University of Ghana, Accra, Ghana.
3. National Dengue Control Unit, Ministry of Health, Colombio, Sri Lanka.
4. Département de Virologie, Institut Pasteur de Dakar, Dakar, Senegal.
5. MRC Centre for Global Infectious Disease Analysis, School of Public Health, Imperial College London, London, United Kingdom.
6. Faculdade de Medicina, Instituto Medicina Tropical, Universidade de São Paulo, São Paulo, Brazil
7. Unité Parasitologie et Entomologie, Département Risques Vectoriels, Institut de Recherche Biomédicale des Armées, Marseille, France.
8. Laboratorio de Biología de Vectores y Parásitos, Instituto de Zoología y Ecología Tropical, Facultad de Ciencias, Universidad Central de Venezuela, Caracas, Venezuela.
9. Institute of Tropical Medicine "Pedro Kouri", WHO/PAHO Collaborating Center for the Study of Dengue and Its Control, La Lisa, Cuba.
10. Departamento de Infectologia, Universidade Federal do Rio Grande do Norte, Natal, Brazil.
11. Directorate General of Primary Health Care, Federal Ministry of Health, Khartoum, Sudan.
12. WHO Collaborating Centre for Vector-Borne Diseases, Department of Epidemiology and Biostatistics, Research Centre for Emerging and Reemerging Infectious Diseases, Pasteur Institute of Iran, Tehran, Iran.
13. Environmental Health Institute, National Environment Agency, Singapore.
14. Centre for Infectious Disease Control, National Institute for Public Health and the Environment, Bilthoven, The Netherlands.
15. Department of Entomology and Nematology, University of California, Davis, California, United States of America.
16. South Delhi Municipal Corporation, Delhi, India.
17. Division of Emerging Viral Threats, One Health Surveillance and Vaccines; and Infectious Disease and Oncology Research Institute, University of the Witwatersrand, Johannesburg, South Africa.

Corresponding author (NRF: nfaria@ic.ac.uk)

Supplementary Figure

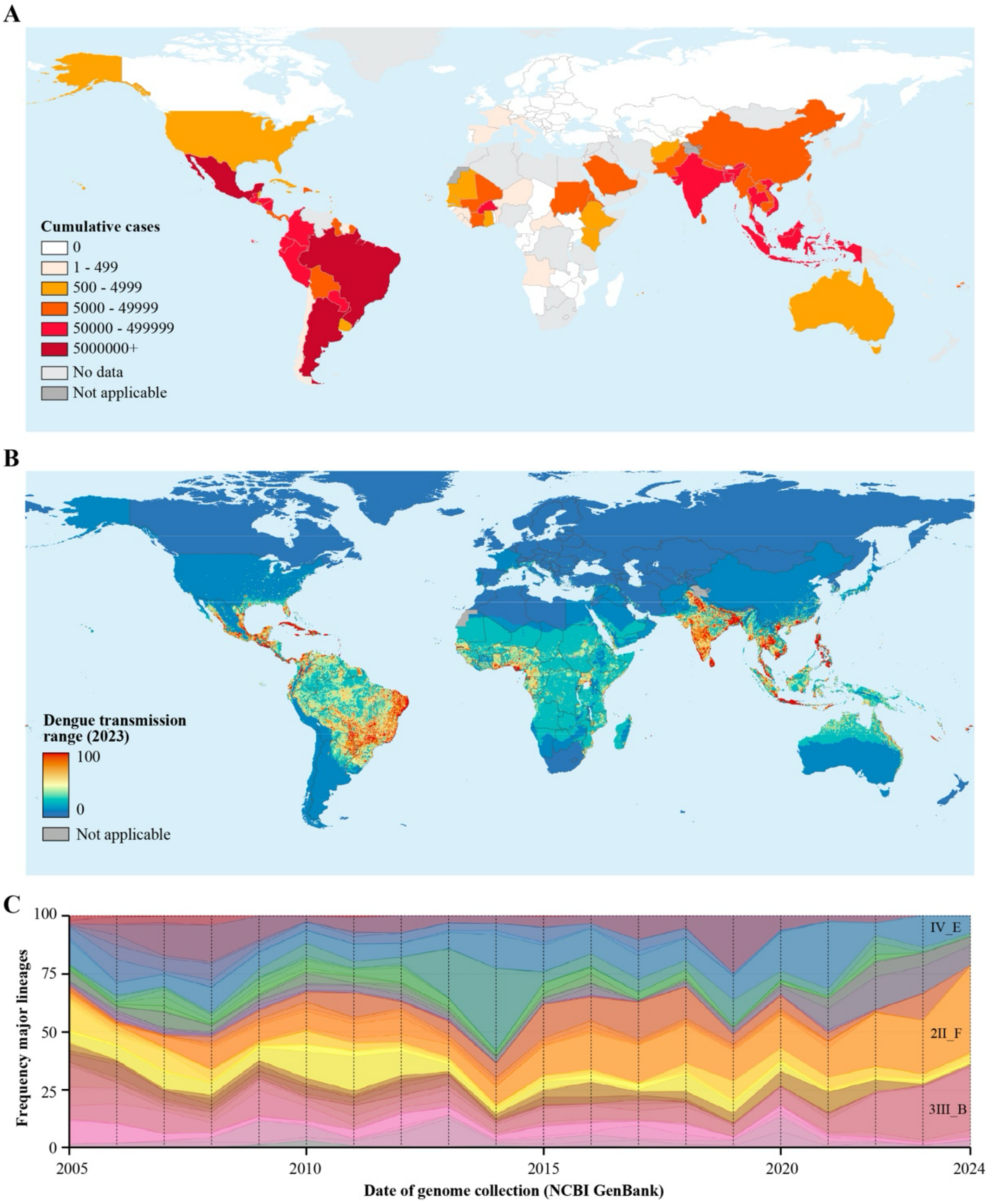

Supplementary Figure. Global distribution of dengue cases, dengue risk, and temporal changes in DENV lineages. (**A**) Global distribution of reported dengue cases, illustrating regions with the highest incidence in 2024. (**B**) Geographic risk map for arboviral diseases, highlighting areas with elevated transmission potential based on recent surveillance data. (**C**) Temporal trends in dengue virus (DENV) lineages, showing the three most prevalent lineages reported globally in 2024: 2II_F (37%), 3III_B (27%), and 1V_E (9%). Data sourced from the WHO dengue dashboard (*https://worldhealthorg.shinyapps.io/dengue_global/)*.[6]
